# Mutation of *smeRv* Renders *Stenotrophomonas maltophilia* Resistant to First-Line Antibiotics Trimethoprim/Sulfamethoxazole and Levofloxacin

**DOI:** 10.3390/antibiotics14060550

**Published:** 2025-05-28

**Authors:** Nuchjaree Boonyong, Nisanart Charoenlap, Parinya Tipanyo, Pitthawat Grittanaanun, Skorn Mongkolsuk, Paiboon Vattanaviboon

**Affiliations:** 1 Program in Applied Biological Science—Environmental Health, Chulabhorn Graduate Institute, Bangkok 10210, Thailand; nuchjaree@cgi.ac.th; 2 Laboratory of Biotechnology, Chulabhorn Research Institute, Bangkok 10210, Thailand; nisanart@cri.or.th (N.C.); parinyati@cri.or.th (P.T.); pitthawatg@cri.or.th (P.G.); skorn@cri.or.th (S.M.); 3 Center of Excellence on Environmental Health and Toxicology (EHT), Office of the Permanent Secretary (OPS), Ministry of Higher Education, Science, Research and Innovation (MHESI), Bangkok 10400, Thailand

**Keywords:** AMR, antibiotic, antimicrobial resistance, co-trimoxazole, enrofloxacin, fluoroquinolone, levofloxacin, *Stenotrophomonas maltophilia*, veterinary antibiotic

## Abstract

**Background:** *Stenotrophomonas maltophilia* is one of the common causative agents of hospital-acquired infections worldwide. The major concern regarding *S. maltophilia* infections is its extreme resistance to multiple antibiotics. **Methods:** Enrofloxacin-resistant mutants of *S. maltophilia* K279a were selected using a serial passage technique. **Results:** In this study, we showed that one of the mutant strains, KE507, which was selected from *S. maltophilia* K279a for its resistance to the veterinary drug enrofloxacin, conferred resistance to trimethoprim/sulfamethoxazole (co-trimoxazole), levofloxacin, and minocycline as per the Clinical and Laboratory Standards Institute guideline. These antibiotics are the first-line drugs routinely used to treat *S. maltophilia* infections. The KE507 mutant also showed increased resistance to all tested quinolones, azithromycin, and neomycin. Molecular characterization using whole genome sequencing, antibiotic resistance gene expression profiles, and mutational analysis indicated that inactivation of SmeRv (Q208insHSPRFTW), a transcriptional regulator of the SmeVWX multidrug efflux pump, contributes to resistance to quinolones (including levofloxacin), co-trimoxazole, and partially to neomycin, but not to azithromycin or minocycline. These data, together with in silico structural analysis, suggest that the mutation of SmeRv causes a conformational change in the SmeRv structure, which leads to the activation of SmeVWX efflux transporter expression and subsequent resistance to co-trimoxazole and quinolone antibiotics. **Conclusion:** *S. maltophilia* can thus acquire resistance to the antibiotics primarily used to treat *S. maltophilia* infections through the mutation of SmeRv.

## 1. Introduction

*Stenotrophomonas maltophilia*, a gram-negative, non-fermentative, aerobic bacillus, is an opportunistic pathogen recognized as one of the leading causes of hospital-acquired infections, particularly in immunocompromised individuals [1]. Risk factors including cystic fibrosis, underlying malignancy, organ transplantation, human immunodeficiency virus infection, prolonged hospitalization, admission to an intensive care unit, mechanical ventilation, immunosuppressive therapy, the prolonged use of antibiotics (especially carbapenems), and the use of indwelling catheters, are known to be associated with acquired *S. maltophilia* infections [2,3]. *S. maltophilia* commonly causes severe pneumonia and bloodstream infections, with mortality rates as high as 56% [4]. *S. maltophilia* infection is of particular concern due to its strong resistance to multiple antibiotics, which makes this infection difficult to treat and has led to its association with high mortality. Although considered a low virulence pathogen, *S. maltophilia* harbors numerous genes required for pathogenesis and antimicrobial resistance [1,5]. It exhibits inherited resistance to a broad spectrum of antibiotics, including fluoroquinolones, β-lactams, cephalosporins, aminoglycosides, macrolides, carbapenems, tetracyclines, chloramphenicol, polymyxins, and sulfonamides [6]. *S. maltophilia* is equipped with an array of genes that potentially contribute to resistance to multiple antibiotics, including those encoding efflux pumps, antibiotic-degrading or -modifying enzymes, and antibiotic resistance proteins [7]. Efflux pumps are known to contribute to the resistance of *S. maltophilia* to broad-spectrum antibiotics. The majority of the key efflux pumps in *S. maltophilia* belong to the resistance-nodulation-cell division (RND) family and include SmeABC, SmeDEF, SmeGH, SmeVWX, and SmeYZ [7,8]. RND efflux pumps, which are tripartite systems composed of inner membrane protein (IMP), outer membrane protein (OMP), and membrane fusion protein (MFP), extrude substrates from the inner leaflet of the inner membrane and cytoplasm directly into the extracellular space [9]. The RND substrates can be different classes of antibiotics. Several *S. maltophilia* RND efflux pumps have been characterized in terms of their contributions to antibiotic resistance and gene regulation. One of the most well-defined RND efflux pumps is SmeVWX, which confers resistance to fluoroquinolones, chloramphenicol, and tetracycline [10]. Overexpression of SmeVWX is associated with trimethoprim/sulfamethoxazole (co-trimoxazole) resistance [11]. The components of the SmeVWX efflux pump are encoded by a 5-gene operon: *smeU1*, *smeV*, *smeW*, *smeU2*, and *smeX* [10]. SmeV, SmeW, and SmeX are the MFP, IMP, and OMP, respectively, while SmeU1 and SmeU2 belong to the family of short-chain dehydrogenases/reductases [10]. The expression of the *smeU1VWU2X* operon is tightly controlled by two LysR-type transcriptional regulators: a divergently transcribed SmeRv [10] and AzoR [12]. Under physiological conditions, *smeRv* is expressed at a low level, whereas a relatively high level of *azoR* is expressed. AzoR functions as a repressor on the *smeU1VWU2X* promoter [12], while SmeRv can act as a negative or positive regulator, likely depending on the absence or presence of an unknown activator ligand [10]. Moreover, under superoxide stress, the expression of the *smeU1VWU2X* operon is upregulated through the function of SoxR [13,14], a MerR-type transcriptional regulator that can sense and respond to superoxide anion [15]. Thus, the expression of SmeVWX is well orchestrated. However, to date, no evidence has been provided to show that antibiotic exposure can induce its expression.

Antibiotic therapy for *S. maltophilia* infection presents a significant clinical challenge due to its multiple antibiotic resistance characteristic. With regard to *S. maltophilia*, the Clinical and Laboratory Standards Institute (CLSI) has defined the breakpoints for trimethoprim/sulfamethoxazole, levofloxacin, minocycline, ceftazidime, cefiderocol, and chloramphenicol [16]. Co-trimoxazole is the first-line treatment for *S. maltophilia* infections, given its high susceptibility rate, while levofloxacin is the second most common therapeutic choice in clinical practice [17].

*S. maltophilia* readily develops strains with increased resistance to multiple antibiotics when exposed to selection pressures, including antibiotics [11,18,19], the biocide triclosan [20], antimicrobial peptides [21], and the agrochemical paraquat [22]. In this study, one of the mutants selected for veterinary antibiotic enrofloxacin resistance showed a resistant phenotype to both co-trimoxazole and levofloxacin, according to the CLSI guideline. Experimental evidence strongly suggests that this resistance arises from a mutation in *smeRv*, which leads to the upregulation of SmeVWX and antibiotic resistance.

## 2. Results

### 2.1. Isolation of the Fluoroquinolone-Resistant Mutants

In an effort to isolate fluoroquinolone-resistant mutants of *S. maltophilia* K279a, enrofloxacin—an antibiotic commonly employed in veterinary medicine—was chosen as the driving force. This antibiotic is frequently utilized in aquaculture, and approximately 70% of administered enrofloxacin enters the aquatic environment [23]. Such exposure impacts environmental and pathogenic microbes, including *S. maltophilia*, as the residual enrofloxacin serves as a selection pressure for antibiotic resistance.

**Figure 1:**
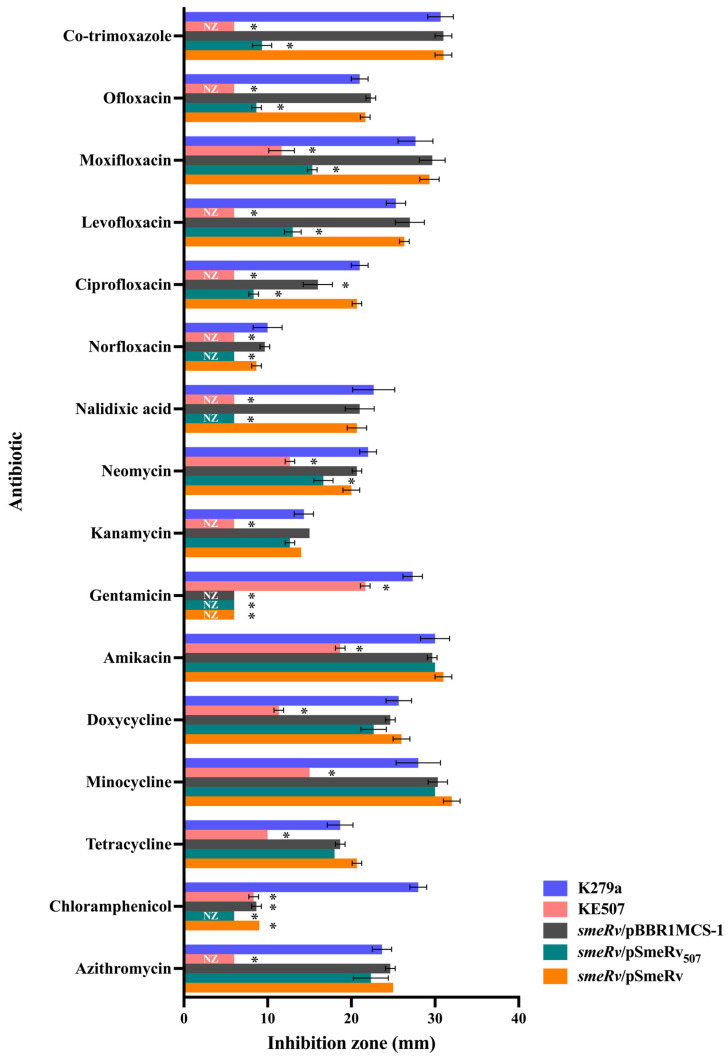
Antibiotic susceptibility profiles in *S. maltophilia* strains. Data present the mean and SD of the inhibition zone diameter (mm) from three independent experiments. An asterisk (*) denotes a statistically significant difference at *p* < 0.05 compared to the K279a wild-type, as determined by the independent samples *t*-test. NZ, no inhibition zone.

Using a serial passage method with increasing concentrations of enrofloxacin, enrofloxacin-resistant colonies capable of growing on plates containing ≥ 4 μg/mL enrofloxacin were obtained. Among these, a particular isolate, KE507, exhibited high-level resistance to multiple antibiotics. Using the standard Kirby–Bauer disc diffusion assay, KE507 showed no inhibition zone (NZ) against nalidixic acid (a quinolone), fluoroquinolones (norfloxacin, ciprofloxacin, levofloxacin, and ofloxacin), co-trimoxazole, and aminoglycoside kanamycin (Figure 1).

KE507 also exhibited significantly reduced inhibition zones compared to the K279a parental strain for aminoglycosides (gentamicin, amikacin, and neomycin), tetracyclines (tetracycline, doxycycline, and minocycline), the fluoroquinolone moxifloxacin, and chloramphenicol. According to the CLSI guideline [16], which provides the breakpoints of inhibition zone sizes for certain *S. maltophilia*-specific antibiotics, KE507 is classified as resistant to co-trimoxazole (breakpoint ≤ 10 mm), levofloxacin (breakpoint ≤ 13 mm), and minocycline (breakpoint ≤ 20 mm).

### 2.2. Mutation Analysis of S. maltophilia KE507

**Figure 2:**
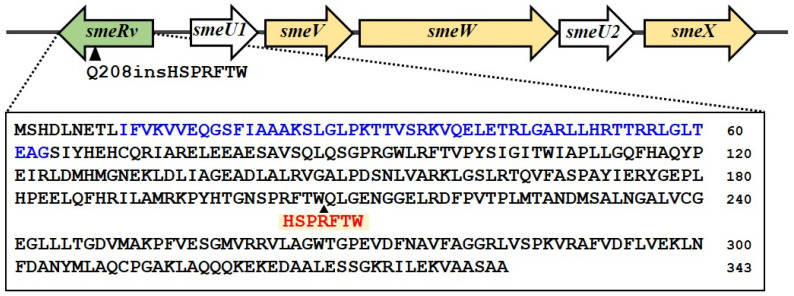
Gene organization at the *smeRv* locus. The *smeRv* is located adjacent to the *smeU1VWXU2* operon in a head-to-head fashion. In the KE507 mutant, the insertion of HSPRFTW occurs at the Q208 residue. The putative DNA-binding domain is highlighted in blue text. The arrowhead indicates the Q208 position.

The whole genome sequence of KE507 was determined and aligned with that of the *S*. *maltophilia* K279a parental strains (GenBank accession no. NC_010943.1) [7]. The identified mutations are summarized in Table 1. A frameshift insertion mutation was observed in *smlt0570* (putative sensor histidine kinase/response regulator fusion protein), where 74 nucleotides were inserted, which caused the Q459fs mutation of Smlt0570. Moreover, an in-frame insertion of GGCATGCCG was detected, which generated Smlt0570 with the H565insAMH mutation. It is thus likely that *smlt0570* was inactivated in KE507. Interestingly, an in-frame insertion of the sequence, TGCCAGGTGAAGCGCGGCGAA, occurred in *smlt1827* (*smeRv*), which led to the generation of SmeRv with the Q208insHSPRFTW mutation (Table 1 and Figure 2). This insertion was confirmed by DNA sequencing of the PCR fragment covering smeRv gene. SmeRv, a LysR-type transcriptional regulator, functions as a transcription regulator of the *smeU1VWU2X* operon, which encodes the SmeVWX multidrug efflux transporter and is located adjacently in a head-to-head fashion (Figure 2). Nonsynonymous and synonymous SNVs were found in *smlt0042* (putative rearrangement hotspot [RHS]-repeat protein containing the DUF6531 domain with an unknown function) and *smlt3968* (conserved hypothetical protein), respectively (Table 1). An SNV (C>A) was detected in the intergenic region between *smlt2891* (putative two-component system response regulator) and *smlt2892* (putative major facilitator superfamily transporter). Since *smlt2891* and *smlt2892* were located in a tail-to-tail arrangement, the SNV occurred in a non-promoter region. No mutations were detected in the *smqnrB* gene encoding protein that diminishes the activity of fluoroquinolone antibiotics and is mainly responsible for fluoroquinolone resistance in *S. maltophilia* [24,25].

### 2.3. Characterization of the Mutations Responsible for the Antibiotic Resistance of KE507

As SmeRv is a transcriptional regulator of the multidrug efflux pump SmeVWX, we investigated the contribution of the mutation in *smeRv* to the antibiotic resistance of KE507. The *smeRv* mutant was constructed by insertional inactivation and complemented with pSmeRv_507_ (to yield *smeRv*/pSmeRv_507_), which expressed SmeRv with Q208insHSPRFTW. The antibiotic susceptibility levels were determined and are shown in Figure 1. The expression of SmeRv_507_ increased the resistance levels of the *smeRv* mutant (*smeRv*/pSmeRv_507_) to quinolones (nalidixic acid, norfloxacin, ciprofloxacin, levofloxacin, moxifloxacin, and ofloxacin) and co-trimoxazole to a level close to that of the KE507 mutant. The *smeRv*/pSmeRv_507_ also partially enhanced the resistance to neomycin. Relative to the KE507 mutant, the expression of SmeRv_507_ failed to increase resistance to aminoglycosides (amikacin and kanamycin), tetracyclines (tetracycline, doxycycline, and minocycline), and the macrolide, azithromycin (Figure 1). This evidence suggests that the mutation of SmeRv (Q208insHSPRFTW) contributes to resistance to quinolones, fluoroquinolones, and co-trimoxazole and partially to neomycin resistance but does not play a role in increased resistance to other drugs, including minocycline, as observed in KE507.

**Figure 3:**
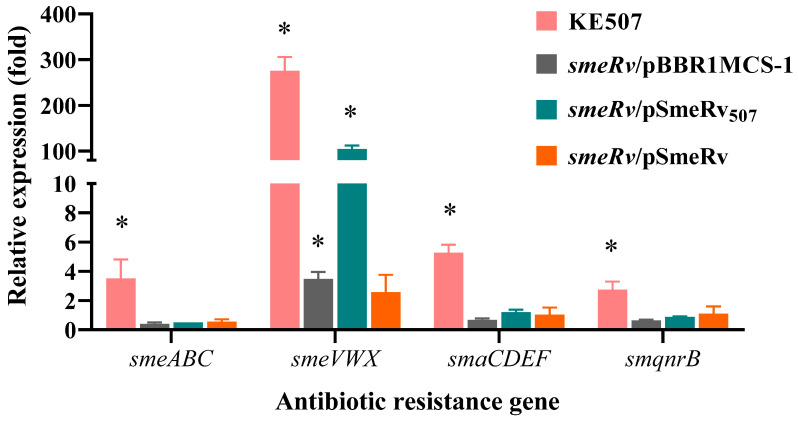
Expression profiles of the antibiotic resistance genes in the *S. maltophilia* KE507 and *smeRv* mutant strains. Data shown are the mean and SD of the relative expression (fold) compared to the K279a wild-type from three independent experiments. An asterisk (*) denotes a statistically significant difference at *p* < 0.05 determined by the independent samples *t*-test.

SmeRv is a transcriptional regulator of the *smeU1VWU2X* operon, which encodes the SmeVWX efflux pump. We therefore hypothesized that a mutation of *smeRv* may affect SmeVWX expression. Real-time RT-PCR was performed to determine the expression level of *smeV* (representative of *smeVWX*) in KE507 and other mutant strains. As illustrated in Table 2, KE507 produced dramatically high levels of the *smeVWX* transcript (275.7 ± 30.2) relative to the K279a control wild-type levels. The expression of SmeRv_507_ in the *smeRv* mutant elevated the level of *smeVWX* expression to 108.1 ± 1.6 relative to that of the K279a control (Figure 3). Thus, SmeRv with Q208insHSPRFTW acted as a transcriptional activator of *smeVWX* expression.

We further determined the expression profiles of the main antibiotic resistance genes in *S. maltophilia* in the KE507 mutant, and the results are shown in Table 2. Among the antibiotic resistance genes tested, KE507 expressed increased levels of *smaCDEF* (5.3 ± 0.6), *smeABC* (3.5 ± 1.4), and *smqnrB* (2.7 ± 0.6). Nonetheless, the complementation of SmeRv_507_ in the *smeRv* mutant was not able to enhance their expression levels (Figure 3), which indicated that the increased expression of these antibiotic resistance genes is independent of the *smeRv* mutation.

Notably, the level of *smeVWX* expression in the complemented *smeRv* mutant (*smeRv*/pSmeRv) was not significantly increased (*p* = 0.154) compared to that of the K279a wild-type (Figure 3). This result indicated that high expression of *smeRv* was unable to induce *smeVWX* expression in the wild-type.

### 2.4. The Impact of the Insertion Mutation on the Protein Structure of SmeRv

**Figure 4:**
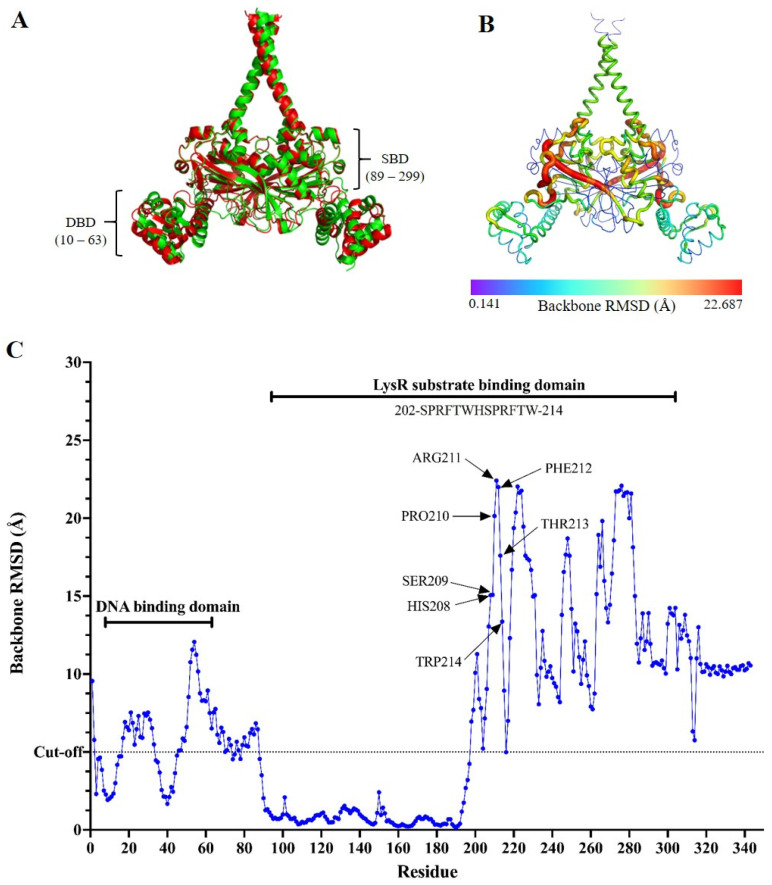
Protein structure modeling of SmeRv and SmeRv_507_. (**A**) Superimposition of the AlphaFold 3-predicted SmeRv model (green) with the SmeRv_507_ model (red) showing an overall RMSD score of 0.801 Å. DBD and SBD represent DNA- and substrate-binding domains, respectively. (**B**) Heatmap of the backbone RMSD value (ranging from 0.141 to 22.687 Å) obtained from the superimposition of the AlphaFold 3-predicted SmeRv and SmeRv_507_ models. (**C**) Backbone RMSD values across the amino acid residue obtained of the superimposed AlphaFold 3-predicted structures of SmeRv and SmeRv_507_. The RMSD cut-off at 5 Å is indicated.

SmeRv is a transcription repressor for the *smeWVX* operon under certain physiological conditions, while SmeRv_507_ acts as an activator. We postulated that the insertion of Q208insHSPRFTW would convert SmeRv_507_ from a repressor to an activator by altering its structure, particularly in the substrate- and DNA-binding domains. As no crystal structure of *S. maltophilia* SmeRv has been resolved, the structures of SmeRv and its variant, SmeRv_507_, were predicted using AlphaFold 3 [26]. The confidence scores (ipTM and pTM) for the SmeRv model structure were 0.81 and 0.8, respectively, while for SmeRv_507_, they were 0.85 and 0.85, which indicated high-quality prediction. The two structures were then superimposed to analyze the structural similarities and differences using the Pymol command line. The overall RMSD between the SmeRV and SmeRv_507_ structures was calculated to be 0.801 Å (Figure 4A), which suggested a relatively small overall structural change. To identify the regions of localized structural differences, a per-residue RMSD analysis was performed, and this revealed that the structural impact of the insertion mutation was not uniform across the protein (Figure 4B,C). The DNA-binding domain (residues 10–63) showed a significant increase in RMSD values from 5.094 to 12.065 Å in the region between residues 17–33 and 46–63. The LysR substrate-binding domain (residues 89–299) exhibited significantly higher per-residue RMSD values, particularly between residues 202 and 214 (SPRFTWHSPRFTW). The peak RMSD value within this region reached 22.411 Å at residue 211 (Arg11). The region between residues 230 and 300 also showed elevated RMSD values ranging from 10 to 20 Å. This indicates that the insertion mutation induced substantial structural changes within the substrate-binding domain. The RMSD values in the loop region (residues 91–194) between the two domains were close to 0, which suggested minimal impact from the insertion mutation.

## 3. Discussion

In this study, *S. maltophilia* enrofloxacin-resistant mutant KE507 was found to be resistant to co-trimoxazole, levofloxacin, and minocycline. Co-trimoxazole is the first-line antibiotic recommended for the treatment of *S. maltophilia* infections due to its high susceptibility rate, while levofloxacin is the second most common therapeutic choice in clinical practice. Minocycline is also used in combination with co-trimoxazole for the treatment of moderate-to-severe *S. maltophilia* infections owing to its promising activity and global susceptibility rate, which exceeds 95% [27,28,29]. Thus, KE507 exhibits extreme resistance to routinely used antibiotic therapies.

The KE507 mutant harbored three mutations that were likely to impact its protein functions: an insertion mutation in *smeRv* (Q208insHSPRFTW), a frameshift insertion mutation in *smlt0570* (putative two-component hybrid regulator), and SNVs in *smlt0042*, which resulted in the generation of the putative RHS-repeat protein with the R631P mutation.

The expression of the *smeU1VWU2X* operon encoding the SmeVWX efflux pump proteins, SmeU1 and SmeU2, is controlled by SmeRv [10]. The components of the SmeVWX efflux pump include the MFP (SmeV), the RND transporter (SmeW), and the OMP (SmeX). These SmeVWX proteins share a high amino acid sequence identity with the *Pseudomonas aeruginosa* MexEF-OprN system [30]. Both SmeU1 and SmeU2 are putative short-chain dehydrogenases/reductases, but their roles in antibiotic resistance are unclear [14]. Notwithstanding, SmeU2 is implicated in alleviating external oxidative stress [14]. Physiologically, SmeRv negatively regulates the expression of the *smeU1VWU2X* operon, as shown by the increase in the operon expression in the *smeRv* mutant (Figure 3), which was in good agreement with a previous report [10]. Under normal growth conditions, LysR-type transcriptional regulators (LTTRs) generally bind to the promoter regions of their target genes. In so doing, they impede the binding of RNA polymerase and thus function as transcriptional repressors. In the presence of effector molecules, the binding of these effector ligands to the effector-binding domain of LTTRs induces a configurational change, and they are converted into activators that facilitate the binding of RNA polymerase [31]. Unfortunately, the effector molecules for SmeRv have not been characterized to date.

The observation that the expression of SmeRv_507_ (SmeRv with Q208insHSPRFTW) could activate the expression of SmeVWX in the *smeRv* mutant (*smeRv*/pSmeRv_507_) strongly suggests that the mutation in SmeRv changed its function from a repressor to an activator. In silico analysis of the putative SmeRv structure revealed a significant alteration in the conformation of the SmeRv Q208insHSPRFTW mutant compared to the proposed wild-type SmeRv structure (Figure 4A–C). We postulated that the insertion of the HSPRFTW sequence at the Q208 residue may have changed the configuration of SmeRv to make it resemble a transcriptional activator, thereby inducing SmeVWX expression. The overexpression of SmeVWX in the KE507 mutant (more than 250-fold relative to the wild-type level) rendered KE507 resistant to co-trimoxazole and levofloxacin and increased its resistance to nalidixic acid and the other fluoroquinolone antibiotics. Furthermore, the expression of the *smeU1VWU2X* operon was negatively controlled by another transcriptional regulator, AzoR (Smlt3089) [10,12]. No mutations were detected in either *azoR* or its putative promoter region.

The superimposition of the predicted structures of SmeRv and its variant SmeRv_507_ showed high RMSD values (ranging from 10 to 22.411 Å) in the substrate-binding domain, particularly at residues 202–214 (SPRFTWHSPRFTW), which suggested that the insertion mutation Q208insHSPRFTW induced significant conformational changes in this region. This may have distorted the substrate-binding pocket and potentially reduced the affinity for the native substrate or altered the substrate specificity. The structural changes in the substrate-binding domain could have disrupted the allosteric communication between this and the DNA-binding domain. This could have impaired the protein’s ability to regulate DNA binding in response to the increased substrate levels. LysR-type proteins often utilize a conformational change in the substrate-binding domain to propagate a signal to the DNA-binding domain, thereby altering its binding characteristics for the target DNA sequence [32,33,34]. For instance, binding of the effector molecules (salicylate) or the H169T mutation in the substrate-binding domain of DntR, an LTTR, induces a more expanded subunit rearrangement, significantly shifting the DNA-binding domain positions and activating its function [35].

We found an alteration in the DNA-binding domain with significantly increased RMSD values between residues 39 and 63 (ranging from 5.094 to 12.065 Å), which indicated a substantial conformational change within this region. The observed conformational changes in both the substrate- and DNA-binding domains would have altered the protein–DNA interactions and facilitated the function of RNA polymerase on the regulated promoters. However, this protein structure modeling analysis would have been more accurate if the crystal structure of SmeRv had been resolved and the known substrates for SmeRv characterized.

The enhanced expression of *smaCDEF* (ABC transporter) and *smeABC* (RND efflux pump) in the KE507 mutant was not a result of the mutation in the *smeRv*. Overproduction of the SmaCDEF efflux pump, which confers resistance to levofloxacin, has been reported in the K279a mutant selected for levofloxacin resistance. The mechanism through which this overproduction occurs is unknown [36].

SmeABC efflux pumps mainly contribute to resistance to fluoroquinolones and aminoglycosides [37]. The expression of the *smeABC* operon is positively controlled by SmeSR, which is a two-component regulatory system [37]. However, the specific stimuli for SmeS activation are currently unknown. Several clinical isolates of *S. maltophilia* that overexpress *smeABC* have been associated with multidrug resistance, especially ciprofloxacin resistance [38,39]. No molecular studies on these isolates have been undertaken, so the involvement of SmeSR in the high expression of *smeABC* is unclear.

Interestingly, the increased resistance to tetracyclines (tetracycline, doxycycline, and minocycline) observed in the KE507 mutant was not due to the elevated expression of the efflux pumps known to contribute to resistance to tetracyclines, including the SmeDEF and SmeIJK RND efflux pumps. The tetracycline resistance mechanisms in *S. maltophilia* are poorly understood [1]. Further investigations are required to identify the mechanisms behind the resistance to tetracyclines in the KE507 mutant.

The two-component hybrid regulators are intricate proteins that possess both a histidine kinase domain and a receiver domain. This group of regulators has garnered significant interest due to their involvement in complex signaling mechanisms [40]. The roles of several hybrid histidine kinase regulators from *P. aeruginosa*, including SagS, RetS, RadS, and PA1611, have been elucidated [40,41,42,43]. While all these regulators are implicated in the regulation of biofilm formation, virulence factors, secretion systems, and cytotoxicity, SagS also contributes to antibiotic resistance by activating multidrug efflux pumps, namely, the MexAB-OprM and MexEF-OprN systems [41], which are homologous to the SmeABC and SmeVWX systems in *S. maltophilia* [30]. However, inactivation of *P. aeruginosa* SagS renders the biofilm more susceptible to antibiotics [43]. Frameshift and in-frame mutations in Smlt0570, a putative two-component hybrid regulator, would inactivate its function. If Smlt0570 is functionally orthologous to SagS, KE507 would be expected to show increased antibiotic susceptibility rather than the observed resistance phenotype. Nonetheless, investigations into the mutation of Smlt0570 and its role in conferring antibiotic resistance, including its increased resistance to tetracyclines, aminoglycosides, and chloramphenicol, as well as the unexplained upregulation of *smeABC* and *smaCDEF* expression, warrant further comprehensive analysis.

Smlt0042 encodes a putative RHS-repeat protein that contains a DUF6531 domain. RHS-repeat proteins are a class of toxins employed by bacteria to facilitate intercellular competition and host invasion [44]. To date, no experimental evidence has suggested an association between RHS-repeat proteins and antibiotic resistance. The R631P point mutation in the RHS-repeat protein of KE507 is thus unlikely to confer drug resistance.

## 4. Materials and Methods

### 4.1. Bacterial Growth Conditions

A clinical strain K279a of *Stenotrophomonas maltophilia* [7], isolated from the blood sample of an elderly male patient undergoing chemotherapy at the Bristol Oncology Unit in 1998, was obtained from Professor Matthew B. Avison, at the University of Bristol, United Kingdom, and used as a parental wild-type. Routinely, a single colony of K279a was used as starting inoculum in the lysogeny broth (LB) and growing at 35 °C for overnight with constant shaking at 180 rpm before being sub-cultured into a fresh LB medium at a starting optical density at 600 nm (OD_600_) of 0.1. Exponential-phase cells OD_600_ of 0.5 were used in all experiments.

### 4.2. Selection of KE507 Mutant

Enrofloxacin-resistant mutants of *S. maltophilia* K279a were selected using a serial passage method as previously described [22] with some modifications. An overnight culture was inoculated into LB medium supplemented with 0.5 µg/mL of enrofloxacin—a sub-minimum inhibitory concentration (MIC) level for K279a against enrofloxacin (MIC 1.0 µg/mL)—at a starting OD_600_ of 0.1 and incubated overnight (24 h). This process was repeated for five consecutive passages, each time using fresh LB medium supplemented with increasing concentrations of enrofloxacin (1, 2, 3, 4, and 5 μg/mL). According to the CLSI guideline, the breakpoint for enrofloxacin is ≥4 μg/mL [16]. Consequently, bacterial cultures capable of growing in enrofloxacin concentrations of 4 and 5 μg/mL were spread onto the LB plates containing 4 μg/mL enrofloxacin. A total of 100 isolated colonies were selected, and their antibiotic susceptibility patterns were assessed.

### 4.3. Molecular Biology Techniques

Molecular biology techniques (i.e., RNA extraction, DNA cloning, agarose gel electrophoresis, Southern blot analysis, polymerase chain reaction (PCR), and *Escherichia coli* transformation and conjugation) were conducted in accordance with established protocols [45]. *S. maltophilia* transformation was performed using electroporation [15].

### 4.4. Antimicrobial Susceptibility Testing

The antimicrobial susceptibility of the *S. maltophilia* strains was determined using the standard Kirby–Bauer disc diffusion method [46]. The following antimicrobial discs (Oxoid, Hampshire, UK) were used: nalidixic acid (NA, 30 μg), norfloxacin (NOR, 10 μg), ciprofloxacin (CIP, 5 μg), levofloxacin (LEV, 5 μg), moxifloxacin (MXF, 5 μg), ofloxacin (OFX, 5 μg), amikacin (AK, 30 μg), gentamicin (CN, 10 μg), kanamycin (K, 30 μg), neomycin (N, 30 μg), azithromycin (AZM, 15 μg), tetracycline (TE, 30 μg), doxycycline (DO, 30 μg), minocycline (MH, 30 μg), chloramphenicol (C, 30 μg), and co-trimoxazole (SXT 25 μg). The data are presented as the mean ± standard deviation (SD) from three independent experiments.

### 4.5. Whole Genome Sequencing and Mutation Analysis

Whole genome sequencing of *S. maltophilia* strains was conducted by Porcinotec (Bangkok, Thailand). Essentially, the quality of the purified genomic DNA of the *S. maltophilia* variant (prepared using a GF-1 Bacterial DNA Extraction Kit; Vivantis, Subang Jaya, Malaysia) was assessed with a DeNovix QFX Fluorometer (Wilmington, DE, USA). The genomic DNA library, with DNA fragments tagged with different sequencing adaptors, was prepared using a QIAseq FX DNA Library Preparation Kit (Qiagen, Hilden, Germany), validated on a QIAxcel Advanced System (Qiagen), and quantified using the DeNovix QFX Fluorometer prior to sequencing with V2 chemistry to generate 2 × 250 bp reads on the MiSeq Sequencing System (Illumina, San Diego, CA, USA). The sequencing reads were processed using Trimmomatic (version 0.39) [47] to remove adapters and filter out low-quality reads with a quality score < Q30. A quality assessment of the sequencing reads was performed using FastQC (version 0.12.1) [48]. The resulting reads were mapped to the reference genome sequence of strain K279a (accession no. NC_010943.1) using the alignment program BWA (version 0.7.17). GATK’s Unified Genotyper (version 3.8.1) was used for single nucleotide variant (SNV)/insertion/deletion (InDel) variant calling, and annotation for SNV/InDel was performed by Annovar [49]. The *smeRv* mutation (Q208insHSPRFTW) in strain KE507 was confirmed by DNA sequencing of the PCR fragment covering the *smeRv* gene. The assembled genome sequences of the *S. maltophilia* K279a variant were deposited under the NCBI BioProject (accession no. PRJNA1231703, BioSample SAMN47217596).

### 4.6. Real-Time Reverse Transcription PCR

The expression levels of the antibiotic resistance genes in the *S. maltophilia* strains were quantified using real-time reverse transcription (RT)-PCR as previously described [18]. Briefly, the total RNA was extracted from the exponential-phase cultures of the *S. maltophilia* strains. After treatment with DNase I, 1 μg of total RNA was used for the reverse transcription using a RevertAid Reverse Transcriptase (Thermo Fisher Scientific, Waltham, MA, USA) and random hexamers following the manufacturer’s recommendations. The synthesized cDNA (10 ng) was used as the templates in the PCR reaction, which consisted of the SYBR Select Master Mix for CFX (Thermo Fisher Scientific) and gene-specific primers (listed in Table 3), and was run on a StepOnePlus Real-Time PCR System (Thermo Fisher Scientific) for 40 cycles. Each cycle consisted of denaturation at 95 °C for 30 s, annealing at 60 °C for 15 s, and extension at 72 °C for 30 s. The 16S rRNA, amplified with the primers BT2781 and BT2782 (Table 3), was used as the internal control for normalization. Data analysis was conducted using the cycle threshold method (2^−ΔΔC(t)^), with results expressed as fold changes in gene expression relative to the level in the wild-type K279a. Each experiment was repeated independently three times, and the results were presented as mean ± SD.

### 4.7. Construction of the smeRv Mutant

The *smlt1827* (*smeRv*) mutant was constructed via insertional inactivation of the target gene using the pKNOCK suicide vector [50]. Briefly, a DNA fragment of the *smeRv* gene was amplified using the *S. maltophilia* K279a chromosome as a DNA template with the primers BT8566 and BT8567 (Table 3). The PCR product was then cloned into the pGEM^®^-T Easy Vector (Promega, Madison, WI, USA) before the *Eco*RI fragment was subcloned into pKNOCK-Gm to generate pKNOCK*smeRv*. This recombinant plasmid was transferred to the *S. maltophilia* K279a by conjugation to generate the *smeRv* inactivation mutant. The *smeRv* knockout mutant was confirmed by PCR and Southern blot analysis.

### 4.8. Construction of pSmeRv and pSmeRv_507_

The *smeRv* gene was PCR amplified from the *S. maltophilia* K279a genomic DNA with the primers BT9444 and BT9445 (Table 3) using Hot-Start Ultra High Fidelity DNA polymerase (Tiangen, Beijing, China). A 1062-bp amplicon was cloned into pBBR1MCS-1 [51], a broad-host-range expression plasmid, and cut with *Sma*I to yield pSmeRv. pSmeRv_507_, which expressed SmeRv with a Q208insHSPRFTW mutation, was constructed using the same strategy as that for pSmeRv, except KE507 genomic DNA was used instead of K279a. The recombinant plasmids were verified via DNA sequencing.

### 4.9. Prediction of SmeRv and SmeRv_507_ Protein Structures

Protein structure predictions for SmeRv and its variant SmeRv_507_, which carried the insertion mutation Q208insHSPRFTW, were performed using AlphaFold 3 [26] to examine the impact of this mutation on the structural conformation of the protein. The prediction quality was assessed using per-structure quality estimation scores (ipTM and pTM), where scores above 0.8 indicate high-quality predictions, scores below 0.6 signify failed predictions, and those between 0.6 and 0.8 denote uncertain predictions [52]. The protein family classification and functional domain prediction were performed using InterPro [53]. The SmeRv_507_ model was superimposed onto the SmeRv model, and the overall RMSD value was calculated using PyMOL software (available at: http://www.pymol.org/pymol (accessed on 28 February 2025)) to evaluate the structural similarity. The structural similarity was quantified by calculating the backbone root mean square deviation (RMSD) for each amino acid residue pair from corresponding chains of proteins to ensure identical residue numbers and comparable conformations. A high RMSD value indicates dissimilarity, while a value of zero means an identical conformation structure. Generally, two proteins with similar structures will have an overall RMSD value of less than 3 Å [54]. In this study, the cut-off backbone RMSD value for protein structural dissimilarity was set at 5 Å [55].

### 4.10. Statistical Analysis

The statistical analysis was performed using IBM SPSS Statistics 26 (SPSS Inc., Chicago, IL, USA). Comparisons of the antimicrobial susceptibility profiles and gene expression levels between the *S. maltophilia* variant strains and K279a wild-type were performed using the independent sample *t*-test. A *p*-value less than 0.05 was considered statistically significant.

## 5. Conclusions

*S. maltophilia* has the potential to acquire resistance to the mainstay antibiotics, co-trimoxazole and levofloxacin, through a mutation in SmeRv, the transcriptional regulator of the *smeU1VWU2X* operon. The insertion of the HSPRFTW sequence at Q208 in SmeRv altered its architecture, particularly at the substrate- and DNA-binding domains, in a way that was possibly similar to its activator configuration, and this likely facilitated the transcription of the *smeU1VWU2X* operon by RNA polymerases. This alteration resulted in the overexpression of the SmeVWX efflux pump and conferred *S. maltophilia* KE507 with high levels of resistance to co-trimoxazole and levofloxacin. Furthermore, KE507 exhibited resistance to minocycline, another antibiotic effective against *S. maltophilia*, through mechanisms that remain unelucidated. This study raises significant concerns about the potential for *S. maltophilia* to acquire resistance to the key therapeutic agents used to manage its infections, particularly when exposed to certain drivers, including residual fluoroquinolone antibiotics in the environment.

## Figures and Tables

**Table 1:** Mutational analysis of *S. maltophilia* KE507.

Locus Tag/Position	Gene Product	Type	Effect	Amino Acid Change
*smlt0042*	Putative rearrangement hotspot (RHS)-repeat protein	SNV	G>C	R631P
*smlt0570*	Putative sensor histidine kinase/response regulator fusion protein	INS	Frameshift insertion >CGCCGCCATGGCGGCCTCGGCCTGGGCCTGGCGATCGTGCAGCAGCTGGTCGAACTGCACGGCGGCACCGTCGC	Q459fs
		INS	In-frame insertion >GGCATGCCG	H565insAMH
*smlt1827*	SmeRv (LysR-type transcriptional regulator)	INS	In-frame insertion >TGCCAGGTGAAGCGCGGCGAA	Q208ins- HSPRFTW
*smlt3968*	Conserved hypothetical protein	SNV	C>G	P56P
Position 2935035		SNV	C>A Intergenic region between *smlt2891* (putative two-component system response regulator transcriptional regulatory protein) and *smlt2892* (putative Major Facilitator Superfamily transporter	-

**Table 2:** Expression profile of antibiotic resistance genes in *S. maltophilia* KE507.

Gene	Locus Tag	Product	Antibiotic Resistance	Relative Expression (Fold)
*smeABC*	*smlt4474-6*	RND-type tripartite efflux protein	β-lactams, Aminoglycosides, Quinolones	3.5 ± 1.4 *
*smeDEF*	*smlt4070-2*	RND-type tripartite efflux protein	Fluoroquinolones, Macrolides, Tetracycline, Chloramphenicol, Cotrimoxazole	0.7 ± 0.1
*smeGH*	*smlt3170-1*	RND-type tripartite efflux protein	Quinolones, Macrolides, Tetracycline, Chloramphenicol	0.8 ± 0.2
*smeIJK*	*smlt4279-81*	RND-type tripartite efflux proteins	Aminoglycosides, Fluoroquinolones, Tetracycline	1 ± 0.1
*smeOP*	*smlt3925-4*	RND-type tripartite efflux transporter	Quinolones, Aminoglycosides, Macrolides, β-lactams, Doxycycline	1 ± 0.1
*smeVWX*	*smlt1830-3*	RND-type tripartite efflux transporter	Quinolones, Cotrimoxazole, Tetracycline, Chloramphenicol	275.7 ± 30.2 *
*smeYZ*	*smlt2201-2*	RND-type efflux protein and MFP	Aminoglycosides, Cotrimoxazole	0.8 ± 0.4
*mdtD*	*smlt3623*	MFS-type transporter protein	Macrolides, Tetracycline	2.2 ± 0.4 *
*mfsA*	*smlt1083*	MFS-type transporter protein	Fluoroquinolones; Aminoglycosides, Cephalosporins	1.2 ± 0.4
*smrA*	*smlt1471*	ABC-type efflux pump	Quinolones, Tetracycline	1.2 ± 0.3
*tcrA*	*smlt1069*	MFS-type transporter protein	Tetracycline	1 ± 0
*macABC*	*smlt1539*	ABC-type efflux pump	Macrolides, Aminoglycosides	1.2 ± 0.4
*smaAB*	*smlt2642-3*	ABC-type efflux pump	Aminoglycosides	2 ± 0.8
*smaCDEF*	*smlt1651-4*	ABC-type efflux pump	Levofloxacin	5.3 ± 0.6 *
*pmpM*	*smlt1381*	MATE-type efflux protein	Fluoroquinolones	1.3 ± 1.2
*blaL1*	*smlt2667*	β-lactamase-L1	β-lactams	1.1 ± 0.2
*blaL2*	*smlt3722*	β-lactamase-L2	β-lactams	0.8 ± 0.2
*aph(3′)IIC*	*smlt2120*	Aminoglycoside phosphotransferase	Aminoglycosides	2.6 ± 0.8
*aac(6′)-Iz*	*smlt3615*	Aminoglycoside 6′-N-acetyltransferase	Aminoglycosides	0.4 ± 0
*smqnrB*	*smlt1071*	Quinolone resistance protein	Quinolones	2.7 ± 0.6 *

Data shown are means ± SD from three replicated experiments. RND, resistance-nodulation-cell division family; MFS, major facilitator superfamily; adenosine triphosphate binding cassette (ABC) family; MATE, multidrug and toxic-compound extrusion family. * Statistically significant difference at *p* < 0.05 tested by two-independent samples *t*-test.

**Table 3:** List of antibiotic resistance gene primers used in this study.

Primer	Sequence (5′→3′)	Gene	Product ^1^
BT2781	GCCCGCACAAGCGGTGGAG	*16S rRNA* forward primer	rRNA
BT2782	ACGTCATCCCCACCTTCC	*16S rRNA* reverse primer	rRNA
BT8127	CCCGCATCAACCTCGACTAC	*smeABC* forward primer	RND
BT8128	CAGCACCTTTACCTGTGCCT	*smeABC* reverse primer	RND
BT8129	CAACGTCACCCTCGGCTATG	*smeDEF* forward primer	RND
BT8130	CGACGCTCACTTCAGAGAACT	*smeDEF* reverse primer	RND
BT8133	CCGATCCACGTCCTGTTCAA	*smeIJK* forward primer	RND
BT8134	GTAGACGTACTCGCCATCCG	*smeIJK* reverse primer	RND
BT8137	GAACTGGACGTGGCTGACTTC	*smeOP* forward primer	RND
BT8138	CAGGTCGATCAGCACTTCGC	*smeOP* reverse primer	RND
BT8139	GGAGTTCACCAAGGTGCGT	*smeVWX* forward primer	RND
BT8140	GAAGTCGACCTTGCCCGAAT	*smeVWX* reverse primer	RND
BT8141	CCAGTGCCGAGTACGAACAG	*smeYZ* forward primer	RND
BT8142	CGCGCATCGACATTGATACC	*smeYZ* reverse primer	RND
BT8149	ATCGGCGTGATCGGATTCAT	*mdtD* forward primer	MFS
BT8150	CCGATGCGCGAGAAAAGATT	*mdtD* reverse primer	MFS
BT7111	TCTGGTACGGATTGGCCTGC	*mfsA* forward primer	MFS
BT7112	CCGACCATCGAAGGCACCAC	*mfsA* reverse primer	MFS
BT8255	CGGGTGATGATGTCTGGCTT	*tcrA* forward primer	MFS
BT8256	TGAAGGTCACATAGCCGACG	*tcrA* reverse primer	MFS
BT8189	GATCCAGGAACTGGCGGTC	*smrA* forward primer	ABC
BT8190	CTGGGCGATGGTGGTGATG	*smrA* reverse primer	ABC
BT8193	CCGAAGCACAGCTGAAAACTG	*macABC* forward primer	ABC
BT8194	GGTACTTGCGGTCGGGGTC	*macABC* reverse primer	ABC
BT8197	TTGCCGAAGTGGATTCGCAG	*smaAB* forward primer	ABC
BT8198	GTGAGACGATGCGGGTGTAG	*smaAB* reverse primer	ABC
BT9036	TCCGATTCCAGTCCCTCGAT	*smaCDEF* forward primer	ABC
BT9037	CGTATCCAGCCCATCGAACT	*smaCDEF* reverse primer	ABC
BT8199	TATGCGTTCGCCTTCCTCAC	*pmpM* forward primer	MATE
BT8200	GCACCAGCGCTTTCAGGATG	*pmpM* reverse primer	MATE
BT8213	GGTCACCTGCTGGACAACAT	*blaL1* forward primer	ENZ
BT8214	CACTTCGCCGTCCATGATGA	*blaL1* reverse primer	ENZ
BT8215	GGCATTGCTGGACAGGCG	*blaL2* forward primer	ENZ
BT8216	GCCCTTGGCAAAGCTGTTCA	*blaL2* reverse primer	ENZ
BT8225	TAATTGCCACCGCCGAAGAA	*aph(3′)IIc* forward primer	ENZ
BT8226	AGTCATCGGCATCCACCAACC	*aph(3′)IIc* reverse primer	ENZ
BT8231	GACGGTTGGTTTCGCTGAAG	*aac(6′)Iz* forward primer	ENZ
BT8232	GCGGAAATAGACGACCCGTT	*aac(6′)Iz* reverse primer	ENZ
BT6866	TCAATGGCGCCACGCTGAAG	*smqnrB* forward primer	ARP
BT6867	TCCAGCGTTACCCGCGAGAA	*smqnrB* reverse primer	ARP
BT8566	ATGCACATGGGCAACGAGAA	*smeRv* fragment forward primer	*smeRv* fragment
BT8567	GCACCATTCAACGCAGACAT	*smeRv* fragment reverse primer	*smeRv* fragment
BT9444	CCAGGATCCCCGACCAT	*smeRv* full-length forward primer	SmeRv
BT9445	CGCTCTTGTTGCAGGCTA	*smeRv* full-length reverse primer	SmeRv

^1^ RND, resistance-nodulation-cell division family; MFS, major facilitator superfamily; adenosine triphosphate binding cassette (ABC) family; MATE, multidrug and toxic-compound extrusion family; ENZ, antibiotic degrading/modifying enzymes; ARP, antibiotic resistance protein.

## Data Availability

All relevant data are contained within the article.

## Abbreviations

The following abbreviations are used in this manuscript:

CLSI	Clinical and Laboratory Standards Institute
IMP	inner membrane
InDel	insertion/deletion
LTTRs	LysR-type transcriptional regulators
NZ	no inhibition zone
RMSD	root mean square deviation
RND	resistance-nodulation-cell division
RT-PCR	Reverse transcription polymerase chain reaction
SNP	Single nucleotide polymorphism
